# High content genome-wide siRNA screen to investigate the coordination of cell size and RNA production

**DOI:** 10.1038/s41597-021-00944-5

**Published:** 2021-06-28

**Authors:** Micha Müller, Merve Avar, Daniel Heinzer, Marc Emmenegger, Adriano Aguzzi, Lucas Pelkmans, Scott Berry

**Affiliations:** 1 Department of Molecular Life Sciences, University of Zurich, Zürich, Switzerland; 2 Institute of Neuropathology, University of Zurich, Zurich, Switzerland

**Keywords:** RNA metabolism, High-throughput screening

## Abstract

Coordination of RNA abundance and production rate with cell size has been observed in diverse organisms and cell populations. However, how cells achieve such ‘scaling’ of transcription with size is unknown. Here we describe a genome-wide siRNA screen to identify regulators of global RNA production rates in HeLa cells. We quantify the single-cell RNA production rate using metabolic pulse-labelling of RNA and subsequent high-content imaging. Our quantitative, single-cell measurements of DNA, nascent RNA, proliferating cell nuclear antigen (PCNA), and total protein, as well as cell morphology and population-context, capture a detailed cellular phenotype. This allows us to account for changes in cell size and cell-cycle distribution (G1/S/G2) in perturbation conditions, which indirectly affect global RNA production. We also take advantage of the subcellular information to distinguish between nascent RNA localised in the nucleolus and nucleoplasm, to approximate ribosomal and non-ribosomal RNA contributions to perturbation phenotypes. Perturbations uncovered through this screen provide a resource for exploring the mechanisms of regulation of global RNA metabolism and its coordination with cellular states.

## Abstract (summary)

**Table:** 

Measurement(s)	nascent RNA • Image • S phase • nucleolus organization • Cellular Morphology • Cell Cycle Phase
Technology Type(s)	metabolic labelling: 5-ethynyl uridine • spinning-disk confocal microscope • supervised machine learning • Image Processing
Factor Type(s)	gene expression
Sample Characteristic - Organism	HeLa cell
Sample Characteristic - Environment	cell culture

Machine-accessible metadata file describing the reported data: 10.6084/m9.figshare.14332916

## Background & Summary

Coordination of RNA transcript abundance with cell size has been observed in diverse organisms such as fission yeast, *C. elegans*, *Xenopus*, rat, mouse and human – both when comparing organs with differently sized cells^1^, and when comparing individual cells in heterogeneous populations^2–4^. Such transcript-abundance scaling is thought to be mediated by increasing RNA production rates in larger cells^5^. However, the underlying mechanism remains elusive.

To identify regulators of transcriptional scaling to cell size, we have conducted an arrayed image-based genome-wide siRNA screen with single-cell resolution in human HeLa cells. This dataset allows for the detection of perturbations that affect global transcriptional rates, while accounting for cell size and cell cycle stage. The screen provides a comprehensive, genome-wide overview of global transcriptional regulation. Our approach contrasts with the vast majority of studies in the field of transcriptional regulation that focus on relative differences in expression between genes and often normalize out any global changes to RNA abundance or production rates.

To measure RNA production rates, we added a synthetic base-analogue 5-ethynyl uridine (EU) to cell culture media 30 minutes before fixation. In this way, EU is incorporated specifically into nascent RNA^6^. We subsequently coupled a fluorophore to the ethynyl-residue using a copper-catalyzed click reaction, enabling visualization of the nascent RNA. Imaging with a spinning-disk confocal microscope at 20X magnification (pixel size 325 × 325 nm), and subsequent quantitative image analysis allowed us to quantify transcription rates in single cells. In addition to segmenting nuclei and cells, we also used the DNA and total protein stains to segment the nucleolus, using a pixel-classification approach. This allowed for the distinction between nucleolar signal and nucleoplasmic signal intensities. This spatial distinction of nascent RNA enables an approximate quantification of Pol I- and Pol II/III-dependent transcripts in nucleolar, and nucleoplasmic regions, respectively.

Cell cycle stage is a fundamental determinant for RNA production rates for two reasons: firstly, because DNA serves as the template for transcription and secondly, because cell size is coordinated with cell cycle stage. It is therefore important to determine cell cycle stage in addition to cell size. While G1 and G2 cells can be distinguished using DNA content (DAPI staining), assigning S-phase cells requires additional information. The gold standard method in image-based analysis is to measure 5-ethynyl-2′-deoxyuridine (EdU) incorporation as a marker for active DNA replication^7^, however this is not compatible with EU-based measurement of nascent transcription because it uses the same click reaction. Instead, we used immunofluorescence to detect proliferating cell nuclear antigen (PCNA), a protein that localizes to active replication foci. Changes in the texture of PCNA staining observed in the nucleus is a hallmark of S-phase cells. Together, DAPI and PCNA staining enabled us to train a random-forest classifier to assign every cell to S-phase or non-S-phase. Non-S-phase cells were then divided into G1 and G2 phases based on DNA content. This approach enabled robust cell cycle classification, allowing us to measure the cell cycle distribution of each perturbation.

Measuring cell volume in high throughput is technically challenging. To approximate cell size in a scalable manner, we therefore quantified the total protein content of each individual cell as an approximation for cell size, as has been used previously^8,9^. In addition, we calculated quantitative measurements of each cell’s population context, such as number of neighbors and local cell density^10^. The quantification of features, ranging from the subcellular scale to the population-context scale, allows for a multivariate analysis of determinants of RNA production rates and cell size.

While the dataset presented here was primarily collected with the purpose of performing a detailed and comprehensive single-cell analysis of transcription rates, and their perturbation in human cells, the dataset contains single-cell-level information about cell and nuclear size and morphology, cell viability, cell-cycle stage, PCNA abundance and localisation, nucleolar morphology, and cell population characteristics – all across perturbations at the genome-wide scale. As such, it is a resource that may be useful for many different biological research questions. The dataset is made available on the Image Data Resource (IDR, idr0093) which facilitates user-friendly access to the images and quantitative data derived from the images^11^.

## Methods

### Cell culture

HeLa Kyoto cells were originally a gift from J. Ellenberg (EMBL, Heidelberg) and were re-derived from a single-cell clone in our lab. This clone has not been authenticated, but was previously tested for identity by karyotyping^12^. Cells were cultured in DMEM supplemented with 10% fetal calf serum (FCS) and 1% GlutaMAX (Online-only Table 1) and were tested for the absence of mycoplasma before use.

### Experimental design

**Fig. 1:**
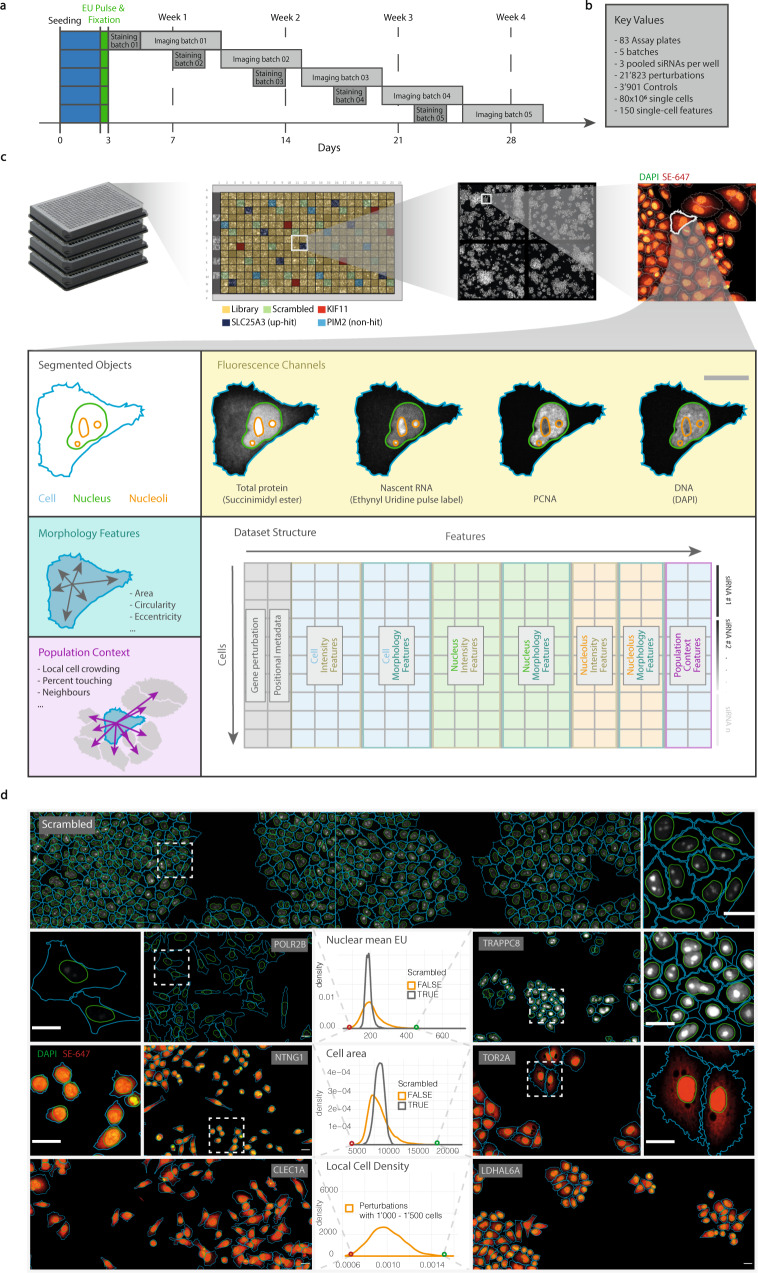
Experimental and computational workflow for primary screen. (**a**) Timeline of experimental workflow. All plates were seeded on the same day and fixed three days later. Staining and imaging were performed in five batches over the following 4 weeks. (**b**) Table of key values (**c**) Overview of arrayed, image-based high-content screen and resulting dataset structure. Plate layout shows pseudo-randomized positions of control wells distributed across the whole plate. Exemplary single cell shown to illustrate segmented objects and image resolution. Three types of features were extracted for the segmented objects: intensity features (from fluorescence channels), morphology features and population context features. The resulting dataset consists of single-cell values as rows, with columns containing feature values and metadata. (**d**) Example images, segmentations, and distributions of quantified features. Plots show the distribution of feature values averaged per-well across all perturbations. Top panel shows a population of cells transfected with scrambled siRNA (negative control) displaying the single-cell heterogeneity in transcription rates. Lower panels show example images of selected perturbations with extreme phenotypes for different features quantified in the screen (left: low, right: high). Gene targeted by siRNA appears on the figure in each case. All images displaying the same channels were scaled identically. Scale bars: 25 µm.

To generate perturbations, cells were reverse transfected in 384-well plates with a pool of 3 independent siRNAs for each gene, using the genome-wide Silencer Select library (Ambion, Thermo Fisher). The library was re-arrayed from its original layout to omit the two outermost columns and rows, due to technical limitations in imaging these plate positions (Fig. 1c). On each plate, there are 22 non-targeting/negative controls (scrambled siRNA), 6 transfection controls (KIF11), 8 gene-targeting but without yielding an EU phenotype (PIM2) and 8 positive controls showing increased EU incorporation (SLC25A3).

All plates of the genome-wide screen were seeded on the same day, in 3 batches. After three days of incubation, plates were pulse-labelled with EU for 30 min, and fixed with paraformaldehyde, taking care that none of the plates had more than +/−1 h deviation from the 72 h incubation with siRNA (Fig. 1a). Fixed cells were then stored in phosphate-buffered saline (PBS) at 4 degrees until further processing, which was performed in 5 batches, each consisting of approximately 18 plates.

### Cell seeding and reverse transfection

5 nl of 5 μM siRNAs from the Ambion Silencer Select library were dispensed with an Echo acoustic liquid handler (Labcyte) into each well of a 384-well plate, resulting in a final siRNA concentration of 5 nM with the final assay-volume of 40 μL.

10 μL of RNAiMax (Thermo-Fisher) at a final concentration of 1:180 in OptiMEM (Gibco) was dispensed into each well containing siRNA and the plate was then incubated at room temperature for 10–60 minutes. Subsequently, 30 μL of cells at a concentration of 26’666 cells/ml (which equals 800 cells per well) were dispensed into the wells in DMEM medium containing 13% FCS and 1.3% GlutaMAX (Online-only Table 1). After allowing cells to settle for 20 minutes at room temperature, plates were then transferred to a Liconics rotating incubator and grown at 37 °C and 5% CO_2_ for 72 h. After the EU pulse (described in detail below), cells were fixed with 4% PFA for 15 minutes at room temperature and subsequently washed with phosphate-buffered saline (PBS). Plates were then stored at 4 °C until proceeding with the staining.

### Metabolic labelling of nascent RNA

Nascent RNA was visualised using metabolic labelling as previously described^6^, with modifications. Briefly, cells were cultured in complete media and pulsed for 30 min with EU at a final concentration of 1 mM, before fixation with 4% paraformaldehyde (PFA) for 15 min. After fixation, cells were permeabilised with 0.5% Triton X-100 and washed 3 times with Tris-buffered saline (TBS) (50 mM Tris pH 8.0, 150 mM NaCl). Click reaction master mix was then prepared as follows: 5 µM Alexa Fluor 488 azide, 2 mM CuSO_4_, 100 mM Sodium ascorbate (Online-only Table 1). All reagents were dissolved or diluted on the day of the screen. The click reaction was added to cells precisely one minute after adding sodium ascorbate to the reaction mix. After 30 min at room temperature, cells were washed 3 times into PBS.

### Immunofluorescence

Cells were blocked in 1% bovine serum albumin (BSA), dissolved in PBS, for 1 h at room temperature on a shaker. After blocking, anti-PCNA antibody was added to cells in 1% BSA, incubated for 2 h at room temperature on a shaker, and subsequently washed with PBS. The secondary anti-rabbit IgG antibody, diluted in 1% BSA, was incubated for 1.5 h on a shaker. Upon washing, cells were incubated with DAPI (dissolved in PBS) for 10 min and then washed and incubated with Succinimidyl Ester coupled with Alexa Fluor-647 (SE) for 10 min (in 50 mM sodium carbonate buffer pH 9.0) (Online-only Table 1). After again washing with PBS, PBS containing Penicillin-Streptomycin was dispensed. All staining and washing steps were performed with a washer/dispenser (BioTek), except for the primary and secondary antibody addition and mixing, which was performed on an Agilent Bravo with 96-well pipette head.

### Imaging

Images were acquired with an automated spinning disk microscope (CellVoyager 7000, Yokogawa) using two Neo sCMOS cameras (Andor, 2,560 × 2,160 pixels), and a 20 × 0.75 NA air objective (Nikon). 9 optical sections were acquired at 3 μm spacing, and were maximum projected before saving. The theoretical axial resolution of the microscope configuration is 1.66 µm at 647 nm, while the average height of HeLa cells is 2.3 µm. Individual cells are therefore typically imaged in 2-3 of the 9 optical sections. DAPI and PCNA (Alexa568) were acquired simultaneously on separate cameras with 405 nm and 561 nm laser illumination for 150 ms per z-section, using BP445/45, BP590/20 filters, respectively. EU (Alexa488) and SE-Alexa647 were acquired simultaneously on separate cameras with 488 nm and 647 nm laser illumination for 200 ms per z-section, using BP525/50, BP675/30 filters, respectively.

### Image processing

Image processing was done using TissueMAPS: a cloud-based, interactive image viewer and analysis tool developed in our lab (https://github.com/TissueMAPS). The pyramid-based visualisation of large datasets, such as the genome-wide screen presented here, allows for fast and easy visual inspection of the images, which facilitates extensive human oversight into artefacts arising from both staining and image analysis, allowing for their detection and exclusion. Furthermore, TissueMAPS handles parallelisation of the computation over a computational cluster, which allows for highly scalable image analysis and feature extraction.

The first step in image processing consists of illumination correction, which corrects shading artefacts derived from the inhomogeneous illumination that occurs in spinning-disk microscopy. This is performed pixel-wise by analysing 20’000 images of each channel^10^. TissueMAPS then stitches the tiled images from each well, and builds an image pyramid for interactive visualisation of the dataset.

Image segmentation and feature extraction is detailed in Table 1. Nuclei and cells were segmented based on DAPI and SE signal intensity, respectively^13^. In addition, nucleoli were segmented based on the pixel probability maps gained from the nucleolus pixel classification (see Nucleoli segmentation). Intensity, texture, area and shape features were extracted from segmented nuclei, cells and nucleoli.

**Table 1:** Image segmentation and feature extraction steps.

Step	Module	Inputs	Output	Function
1	Smooth	DAPI	O1	Smooth DAPI (Gaussian filter)
2	Threshold	O1	O2	Threshold smoothed DAPI to detect nuclei
3	Fill	O2	O3	Fill the binary mask of detected nuclei, to remove any holes in nuclei
4	Separate clumps	O3, DAPI	O4	Cut double nuclei (especially important in regions with high cell crowding)
5	Filter	O4	O5	Filter out objects which are too small or big to be nuclei
6	Label	O5	O6	Label the nuclei mask
7	Register objects	O6	Nuclei	Register the nuclei objects to use for feature extraction further down
8	Smooth	SE	O7	Smooth SE (Gaussian filter)
9	Segment secondary	O7	O8	Use the segmented nuclei as a seed to expand the secondary object (whole cell) from, based on the SE staining
10	Register objects	O8	Cells	Register the cell objects to use for feature extraction further down
11	Smooth	Nucleolus pixel classifier	O9	Smooth the pixel probability maps which were computed with Ilastik
12	Threshold	O9	O10	Threshold smoothed pixel probability maps to detect nucleoli
13	Fill	O10	O11	Fill the binary mask of detected nucleoli, to remove any holes in nucleoli
14	Filter	O11	O12	Filter out objects which are too small or big to be nucleoli
15	Label	O12	O13	Label the nucleoli mask
16	Combine masks	O13 and Nuclei	O14	Make sure that only nucleoli which are inside of the nuclear mask are retained
17	Register objects	O14	Nucleoli	Register the nucleoli objects to use for feature extraction further down
18–40	Measure	DAPI/SE/EU/PCNA	Feature values	Measure morphology, intensity and texture features for the available channels in the objects segmented above

TissueMAPS allows iterative supervised machine learning to classify cells. That is, a particular subset of cells, such as mitotic cells, can be labelled by the user and used as training data for a classifier (support vector machine), which is then applied to all other cells. This function was used to classify mitotic and apoptotic cells and to classify obvious segmentation artefacts (polynucleated cells/incorrectly split nuclei) which were removed from the data for downstream analysis (see Data clean-up).

### Cell-cycle classification

**Fig. 2:**
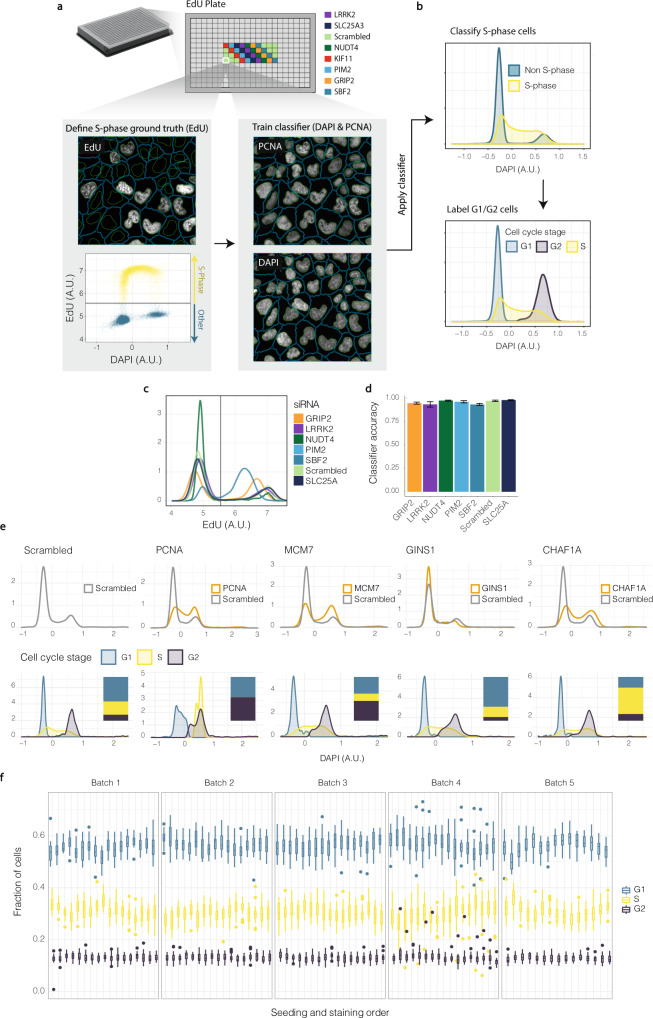
Cell-cycle classification. (**a**) EdU plates in each staining batch were used to train random-forest classifiers to detect cells in S-phase. Ground truth S-phase cells were defined as cells with sum nuclear EdU level above a manually selected threshold. DAPI and PCNA features were used to train the classifier. (**b**) Trained classifiers were then applied to all non-EdU plates to predict S-phase cells. The remaining non-S-phase cells were split into G1- and G2-phases by taking the central local minimum of the (non-S-phase) DAPI distribution as a threshold. (**c**) EdU distributions for all perturbations included in training the S-phase classifier. (**d**) Classifier accuracy (fraction of cells correctly classified in non-training data) for all perturbations included on the EdU plates. Plot shows mean +/− standard deviation across the five staining batches. (**e**) Perturbations previously shown to affect cell cycle distribution. Upper panels show distributions of sum nuclear DAPI intensity in comparison to scrambled siRNA controls. Lower panels show the DAPI distributions divided into cell-cycle stages. Inset stacked bar plots show the proportion of cells in the different cell-cycle stages. (**f**) Fraction of cells in each cell cycle stage for all scrambled siRNA control wells of the screen. Boxplots aggregate values from a single plate and indicate median and interquartile range (IQR), with the upper/lower whisker extending to ±1.5 × IQR.

Supervised machine learning was used to classify cells into S-phase and non-S-phase based on nuclear intensity and texture of Proliferating Cell Nuclear Antigen (PCNA) and DAPI staining (Fig. 2). To generate a ground-truth for training this classifier, we included a separate plate in each staining batch, in which cells were incubated with EdU instead of EU. For these plates, staining was performed identically to the EU-plates, but positive EdU incorporation instead reveals cells undergoing DNA replication rather than transcription^7^. Since we wanted to apply this classifier to perturbed cells, we also included several siRNA perturbations in the training data for this classifier. These were chosen to have perturbed cell cycle distribution, as well as diverse cell and nuclear morphologies that may influence the detection of bona fide S-phase cells. For cells incubated with EdU, we defined S-phase cells as those with sum EdU intensity above a manually selected threshold (Fig. 2a). This data was used as a ground-truth on which the random forest classifier was trained. To assign the remaining cells to either G1 or G2 phase, histograms of the sum nuclear DAPI intensity were plotted for all interphase cells of the entire plate. Cells classified as S-phase were removed and the minimum between the two peaks was used to classify the populations on the lower side as G1 and on the upper side as G2 (Fig. 2b). The S-phase classifier showed an accuracy of 0.97 for scrambled siRNA control wells (on data omitted from the training set), with other perturbations having similar accuracy (Fig. 2c,d).

### Nucleoli segmentation

**Fig. 3:**
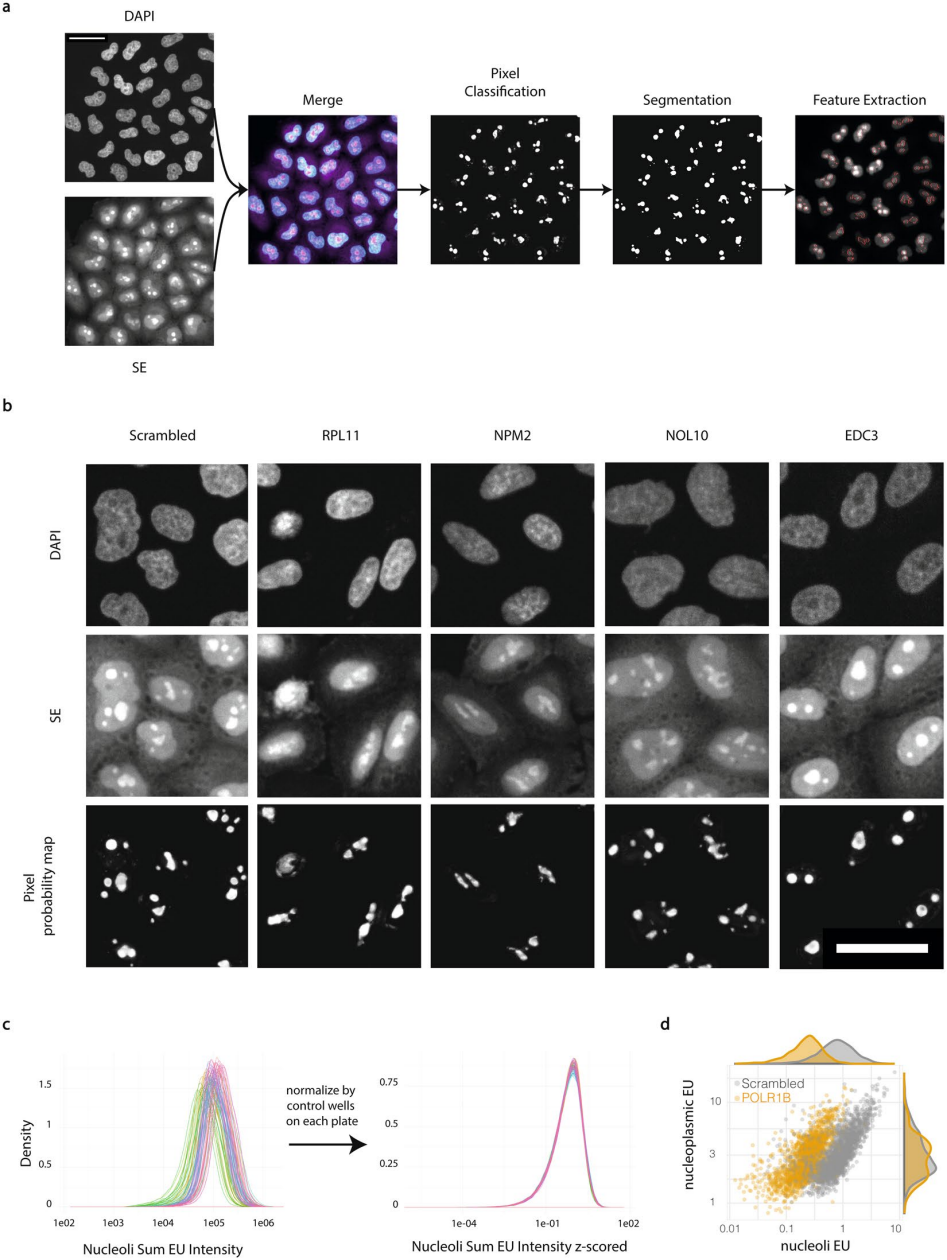
Nucleolus segmentation. (**a**) DAPI and SE images were used together to manually train a two-class pixel classifier for regions of high protein intensity in the nucleus. Resulting pixel probability maps were then thresholded to segment this region as the ‘nucleolus’. Scale bar: 25 µm. (**b**) Perturbations affecting nucleolar morphology and abundance. Representative images of DAPI and SE stains and the pixel classification probability maps for nucleoli in perturbations known to affect nucleolar morphology and number. All images displaying the same channels were scaled identically. Scale bar: 25 µm. (**c**) Histograms of sum EU intensity in the nucleolus per cell, before and after normalization. Normalization was performed by calculating the mean and standard deviation of values for all cells from scrambled siRNA control wells on each plate and using these to standardize the raw values. (**d**) Single-cell sum intensities of EU in the nucleolus and nucleoplasm (non-nucleolus) for wells transfected with POLR1B siRNA or scrambled siRNA.

To approximate the proportions of RNA Polymerase I (RNA Pol I) and RNA Pol II/III-dependent nascent RNA represented by the global transcriptional rates measured here, we employed a pixel-classification approach to segment nucleoli. This allows us to distinguish between nascent ribosomal RNA and non-ribosomal RNA, transcribed in the nucleolus and the nucleoplasm, respectively. To achieve this nucleolar segmentation, we trained a two-class pixel classifier using Ilastik^14^. The classifier uses the information from the DNA (DAPI), which is less intense in the nucleolus, and protein (SE) staining, which is more intense in nucleolar regions (Fig. 3a). We found that including both channels was more robust than SE alone. To ensure robustness across the screen, we trained the pixel classifier using images from different plates of the screen, as well as using images from perturbations that are known to affect nucleolar structure and morphology. This led to robust segmentation of nucleoli, even in perturbations known to lead to strongly perturbed nucleolar morphologies, such as RPL11^15,16^ or NPM2^17^ (Fig. 3b). As proof-of-principle of the utility of this approach, we calculated the nucleolar and non-nucleolar EU intensities, and found that perturbation of POLR1B (RNA Pol I subunit) specifically affects nucleolar (but not nucleoplasmic) RNA production (Fig. 3d).

Despite the robustness of this nucleolar segmentation, we still detected plate-dependent bias in nucleolus size. We hypothesize that this is due to technical variability in SE staining. For this reason, all nucleolar features, including intensity and morphology features, should be normalized to the scrambled siRNA control wells on each plate (Fig. 3c). We found that standardizing relative to scrambled siRNA controls (subtracting the mean and dividing by the standard deviation) eliminated plate-specific differences, allowing for comparisons of nucleolar features across the whole screen.

### Data clean-up

All cells touching a border of the imaging site were excluded. Furthermore, a classifier was trained to classify mitotic and very early G1 cells (non-transcribing) which were excluded from this analysis. This classifier used DAPI texture features as input and also led to exclusion of dead cells and debris that showed similar DNA condensation as mitotic cells.

To exclude cells with segmentation errors, three steps were performed: First, a classifier was trained to classify missegmented cells, using morphology and DAPI intensity features. Second, cells which were quantified to have more than 20% of their DAPI signal in the cytoplasm were removed. Third, cells in which the DAPI signal was more than 4 standard deviations above the mean were excluded (Table 2).

**Table 2:** Number of cells removed at each stage of data clean-up.

Clean-up stage	Number of cells
Total cells	82’098’287
Acquisition errors	76’662
Border cells	10’226’384
Cytoplasmic DAPI	465’726
DAPI outliers	469’935
Missegmented	3’778’709
Mitotic and apoptotic	3’109’692
Cells remaining after clean-up	63’971’179

**Fig. 4:**
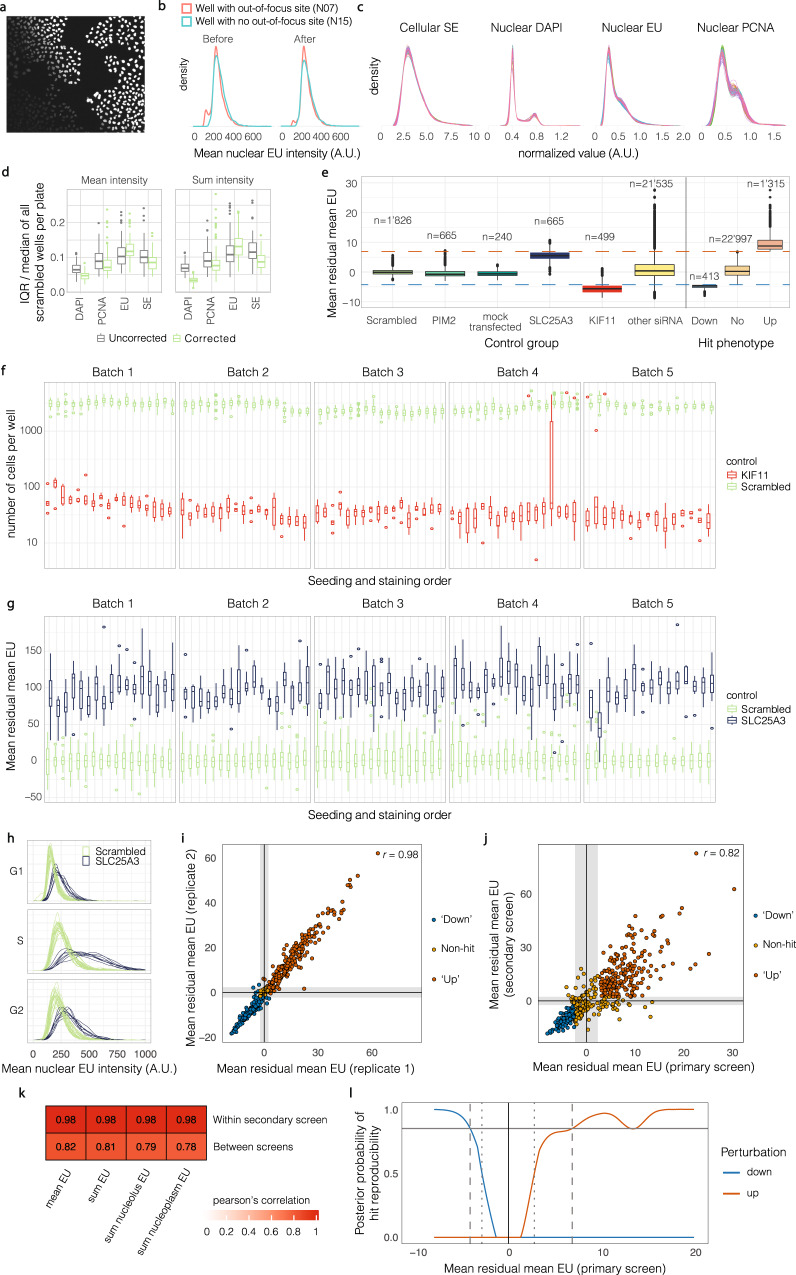
Correction of imaging artefacts and reproducibility of RNA production rate perturbations. (**a**) Example site from a scrambled siRNA control well on plate 9 showing a site identified as out-of-focus. DAPI shown in grayscale. (**b**) Single-cell mean EU intensity distributions before and after removal of out-of-focus sites. Density plots summarise single-cell values from exemplary scrambled siRNA control wells on plate 9. (**c**) Comparison of single-cell sum intensity distributions on each plate after data clean-up and normalization. Each line represents a plate. Scrambled siRNA controls only. (**d**) Well-to-well variability in intensity features on the same plate for scrambled siRNA controls, before and after correction for row/column staining biases. Quantified as the interquartile range (IQR) of well-medians on a plate, normalized by the plate-median. Boxplots summarise values from each plate. (**e**) Mean residual mean EU (see RNA production rate phenotypes) for controls and annotated hits. Boxplots summarise values from each well. Horizontal lines represent hit thresholds. (**f**) Number of cells per well for scrambled siRNA and KIF11 siRNA controls. Boxplots summarise values from each plate. (**g**) As in f, for mean residual mean EU for scrambled siRNA and SLC25A3 siRNA controls. (**h**) Single-cell mean nuclear EU intensity distributions for scrambled siRNA and SLC25A3 siRNA controls on an exemplary plate, by cell cycle stage. Density plots per well. (**i**) Comparison of mean residual EU between replicates of the secondary screen. Shaded regions depict the 1st and 99th percentiles of mean residual EU distributions of scrambled siRNA controls. (**j**) As in I, comparing the secondary screen and genome-wide screens. (**k**) Pearson’s correlations of EU phenotypes within the secondary screen and between the two screens. (**l**) Posterior probability of mean residual mean EU hit reproducibility as a function of mean residual mean EU in the primary screen. Blue line is the posterior probability of a perturbation being a reproducible down-hit, and the red line to being a reproducible up-hit. Dotted and dashed lines represent the thresholds of 50% and 85% posterior probability, respectively. Boxplots indicate median and interquartile range (IQR), with the upper/lower whisker extending to ±1.5 × IQR.

Due to autofocus issues while imaging, a few sites were partially out of focus (Fig. 4a). We found that these cases could be identified in an automated manner, in the following way: we first calculated the means of the single-cell DAPI and PCNA mean intensity measurements per image in each well. We then calculated the difference of the smallest image-mean to the largest image-mean per well. If this difference was higher than a manually selected threshold, the well was labelled as a ‘z-range affected well’. From these wells, all images which were far above or below the median site value (per well) for both DAPI and PCNA were excluded from downstream analysis. Overall, this procedure removed 0.3% of all sites, affecting 2% of all wells. The calculated EU phenotypes (see RNA production rate phenotypes) are based on pooling cells from scrambled siRNA control wells across the whole screen. Because the procedure removed less than 0.15% of imaging sites from these scrambled siRNA control wells, it did not have any effect on the overall variability of EU phenotypes for scrambled siRNA controls. For scrambled siRNA control wells, we verified that single-cell intensity distributions between affected and non-affected wells on the same plate are more similar after this correction (Fig. 4b). All annotations corresponding to excluded data are provided with the single-cell measurements and summary files in the data record.

### Data normalisation

Intensity features were background subtracted using a channel-specific constant value. Intensity features for DAPI, PCNA and SE were then corrected for row and column staining biases using the following equation,

$$I{({\rm{corrected}})}_{p,r,c}=I{({\rm{raw}})}_{p,r,c}+2{I}_{p}-{I}_{p,r}-{I}_{p,c}$$

where *I*_*p*_, *I*_*p,r*_, *I*_*p,c*_ are the median of all cells in plate *p*, or row *r* and column *c* on plate *p*, respectively. This led to decreased variability in these stains between replicate wells on the same plate (Fig. 4d). Applying this same correction to EU intensities led to increased variability between replicate wells on the same plate, so EU values were left uncorrected. This may be because the true differences in EU intensities induced by perturbations exceed the technical variability introduced by row and column effects of EU-staining. For the secondary screen, row/column correction was not applied to any features because it led to increased variability between replicate wells of a plate for almost all features.

Finally, to correct staining differences between plates, we performed plate-wise median rescaling using scrambled siRNA controls. Specifically, we calculated the median intensity values of all cells in scrambled siRNA control wells on each plate, after excluding outlier wells using a plate-wise boxplot rule (+/− 1.5 interquartile range (IQR)). We then multiplied all intensity values on each plate by a correction factor to equalise the plate-wise medians of controls. After this correction, the distributions of cell and nucleus intensity feature values between plates were highly similar (Fig. 4c). For nucleolus features, we found that this median-rescaling was not sufficient to remove plate-specific biases, and additional normalisation was used (see Nucleoli segmentation).

### RNA production rate phenotypes

To quantify RNA production rate phenotypes relative to unperturbed cells, we calculated the mean of all single-cell mean nuclear EU intensities for cells in scrambled siRNA control wells, for each cell cycle phase. We then subtracted these cell-cycle-specific values from the mean nuclear EU intensities of each cell in all perturbation conditions to calculate a ‘residual mean EU’ measurement for each cell. This quantifies the difference in mean nuclear EU intensity of each cell compared to an unperturbed cell in the same cell cycle stage. We reasoned that mean nuclear EU intensity indirectly accounts for cell size due to the scaling of nuclear size with cell size^18^. We then calculated the mean of these residuals for each well as a measurement of the amount that RNA production differs from unperturbed conditions. We refer to this as the ‘mean residual mean EU’. A negative value indicates that a perturbation leads to decreased RNA production rate, on average, compared to scrambled siRNA controls, while positive values indicate an increased RNA production rate.

We used a similar procedure to calculate a quantitative RNA production rate phenotype that is directly corrected for cell size differences. Because HeLa cells are much flatter than they are high (mean cell height 2.3 µm, mean equivalent cell diameter 33.4 µm), protein content measured as sum SE-Alexa647 signal from maximum-intensity projected images is proportional to cell volume^9^. We therefore used this as a measure of cell size. Briefly, we fit a linear regression model to predict sum nuclear EU intensity in cells from scrambled siRNA control wells, using cell size and cell-cycle stage as predictors. We then applied this single-cell linear regression model plate-wise to all cells in the screen. The ‘residual sum EU’ per cell is then calculated as the observed sum nuclear EU signal minus the value predicted by the model trained on negative control cells. We then averaged these single-cell values in each well to yield a quantification of the average deviation in sum nuclear EU signal of a perturbation, compared to a cell of similar size and cell cycle stage from a scrambled siRNA control well. Using these two approaches allows us to account for changes in cell-cycle distribution and/or cell size that may indirectly affect RNA production rate.

### Secondary screen perturbation selection

To select putative hits for secondary siRNA screens, we considered perturbations with ‘mean residual sum EU’ phenotypes below the 1st percentile, or above the 99th percentile for all scrambled siRNA control wells. We focused on genes with at least one gene ontology (GO) annotation, and at least 500 cells. To maintain functional diversity in this panel, we clustered putative hits based on semantic similarity of GO annotations, using R packages GOSemSim and apcluster. To maintain phenotypic diversity in this panel, we clustered single cells using a self-organising map (SOM), and measured a ‘perturbation phenotype’ as the occupancy of cells across the SOM nodes for each perturbation^13^. Functional annotation clusters with more than 10 genes were further subdivided into ‘phenotype clusters’ using k-means. This resulted in ~200 clusters with diversity in both functional annotation and cellular phenotypes. One or two genes were then sampled from each of these clusters, with individual perturbations prioritised using GeneWalk^19^ scores and phenotype strength. This panel was further supplemented with a selection of unperturbed and manually selected perturbations resulting in selection of ~237, 136, 66 putative up, down and non-hits. These represent 4.9%, 6.1% and 0.4% of the viable putative up, down and non-hits, respectively. The overall distribution of RNA production-rate perturbation strengths for the secondary screen was similar to that observed in the genome-wide screen, with high density around the 1st and 99th percentiles of the EU phenotype distributions of negative controls.

### Hit scoring

The RNA production-rate phenotypes ‘mean residual mean EU’ and ‘mean residual sum EU’ were measured in the same manner for the primary and secondary siRNA screen. We first annotated putative hits as “up” and “down” based on 1st and 99th percentiles of distributions of scrambled siRNA control wells in both screens, however this preliminary annotation only takes into account the variability of negative control wells and not the variability of perturbations. To calculate the probability of a gene being consistently annotated as an “up” or “down” hit again upon retesting (i.e. a ‘reproducible’ hit), we combined the results of both screens to calculate the posterior probability *P*(*c*|*v*) where $c\in \{-1,0,1\}$ is −1 for a reproducible “down” hit and +1 for a reproducible “up” hit, and $v\in R$ is the quantitative value of the RNA production rate phenotype from the primary screen (either ‘mean residual mean EU’ or ‘mean residual sum EU’). Hits were defined as reproducible ($c=\pm 1$) if they were classified as up or down, respectively, in both the primary and secondary screen, and 0 otherwise. We then calculated the posterior probability as a function of *v* for $c\in \{-1,0,1\}$ using Bayes’ rule:

$$P(c\,{\rm{| }}\,v)=\frac{P(c)\cdot P(v\,{\rm{| }}\,c)}{P(v)}.$$

For example for *c* = 1, we estimate the likelihood, *P*(*v*|*c* = 1) using a gaussian kernel density estimate of the distribution of primary phenotypes *v* where *c* = 1; the evidence *P*(*v*) as the unconditional kernel density estimate of primary phenotypes (for genes assessed in both screens); and the prior *P*(*c* = 1)= # putative “up” hits/# conditions assayed. Assuming the subset of genes tested in both the primary and secondary screen is not biased to be more or less reproducible, the probability *P*(*c*|*v*) of a hit being reproducible can then be calculated for up and down hits using only *v*, the phenotype from the primary screen (Fig. 4l). These posterior probability distributions can be used to select hit thresholds, as illustrated in Fig. 4l for *P*(*c*|*v*) > 0.5, 0.85. In the data record, we used a cutoff of 0.85 to assign categorical phenotypes. After excluding controls and conditions with low viability (less than 500 cells), we assigned 413 ‘down’ hits and 1183 ‘up’ hits using this cutoff.

## Data Records

Images for the genome-wide screen can be found at the Image Data Resource (IDR: idr0093, 10.17867/10000157), together with quantitative measurements and classifications derived from the images^11^. The data record comprises raw images (DAPI, EU, PCNA, SE channels) for all 83 plates, together with a library file containing the plate layout, gene symbols, siRNA information, and other metadata. We also provide a processed summary file with well-aggregated results. In addition, we provide raw single-cell feature values, and classifier results extracted directly from TissueMAPS (one csv file per plate). We also provide a set of files containing single-cell intensity measurements that have been corrected for staining biases as described in “Data normalisation” (one csv file per plate). Raw single-cell feature files contain data for all non-border cells, together with the classifiers. Processed (corrected) single-cell data files omit cells from the following categories: mitotic/dead, cytoplasmic-DAPI, extreme-DAPI, missegmented, and out-of-focus – as described in “Data cleanup”. The data record also contains the settings files used to process images using the open-source TissueMAPS software. Additionally, the Ilastik project that we used to perform nucleolar pixel classification is also available on IDR. Finally, an example Jupyter Notebook, which can be run remotely on Binder, demonstrates for each plate how to combine the various data sources and make some exploratory plots.

## Technical Validation

### Scrambled siRNA as negative control

To ensure that the scrambled siRNA used did not affect EU incorporation, we compared scrambled siRNA- and mock-transfected wells (containing transfection reagent but no siRNA). A total of 240 mock transfections were included in the genome-wide screen – spread across 4 out of the five staining batches. Additionally, each plate contained 8 wells transfected with siRNA targeting PIM2, which did not yield an EU phenotype (Fig. 1c). The distribution of ‘mean residual mean EU’ values for both mock-transfected and PIM2 siRNA-transfected wells are very similar to scrambled siRNA-transfected wells (Fig. 4e). Overall, this suggests that siRNA transfection *per se* has no effects on the EU phenotypes measured and that the scrambled siRNA used here is an appropriate negative control. We do not account for off-target effects of other siRNAs, which are an inherent property of RNAi perturbations screens^20,21^. In the data record, each well of the genome-wide screen is annotated with the siRNA sequences, which can be used for a more systematic detection of potential off-target effects^22,23^.

### Uniformity of replicate controls in genome-wide screen

The pseudo-randomized plate layout allows for detection and correction of plate-effects. The high number of non-targeting (scrambled siRNA) controls on each plate allow for reliable normalization per plate (Figs. 1c, 4c). KIF11 siRNA served as a transfection control, because knockdown of KIF11 leads to cell death. Cell numbers in wells transfected with KIF11 and scrambled siRNA were consistent across all plates, with the exception of a few wells in batches 4–5 that show increased cell numbers for KIF11 controls (Fig. 4f). This was due to a temporary liquid handling error during the dispensing of the transfection reagent in several columns in plates 62, 65, 67, 68, 69, 70, 71. The error was manually detected during the experiment, and may lead to false negatives on these plates. SLC25A3 serves as a positive control for increased EU incorporation, and was identified during a pilot screen for this project. When targeting SLC25A3, mean residual mean EU is consistently higher than negative control wells on all plates (Fig. 4g,h), demonstrating the reproducibility of the EU phenotypes within and between plates.

### Statistical quality metrics

RNAi screens can be compared using statistical quality metrics such as the Zʹ factor or the strictly standardized mean difference (SSMD)^24^. Whereas Zʹ factor is commonly used for comparing strong positive controls to negative controls, the SSMD allows for interpretation of the quality of positive controls with weak or intermediate phenotype strengths^24,25^. The positive control included in this screen (SLC25A3) shows a weak to intermediate increased EU incorporation phenotype when compared to the range of observed perturbations (Fig. 4e), with only a low percentage of SLC25A3 controls finally annotated as hits (19.8% annotated as up-hits, 80.2% as non-hits). Comparing SLC25A3 and scrambled siRNA controls, SSMD = 2.94, which is an ‘excellent’ value for a moderate up-hit control and a ‘good’ value even if SLC25A3 is regarded as a strong up-hit control^25^.

### Robustness of cell cycle classification

After applying the cell-cycle classification (Fig. 2), we calculated the percentage of cells in each cell cycle phase across the screen. For wells transfected with scrambled siRNA, this was 56% G1, 31% S, 13% G2, in agreement with previous values for unperturbed HeLa cells^26^. The variability in the cell-cycle distribution between wells, and staining batches was small (Fig. 2f), indicating that the classification is reproducible. We found that cells treated with siRNA targeting PCNA showed a very low number of cells assigned to S-phase, illustrating that this approach crucially depends on PCNA staining. Although this is expected on technical grounds, it is likely that PCNA depletion disrupts S-phase progression (Fig. 2e), given its important role in the DNA replication machinery. Finally, we verified that the cell-cycle classification reveals the expected perturbations of the cell cycle as previously observed (Fig. 2e). This was indeed the case for genetic perturbation of MCM7 (G2/M arrest^27,28^), GINS1 (enrichment in G1 phase^29–31^), and CHAF1 (decreased DNA replication rates and therefore prolonged S-phase^32–34^).

### Reproducibility of EU phenotypes

To test for the reproducibility of the quantitative RNA production rate phenotypes, we performed a secondary screen with a targeted subset of hits (see Secondary screen perturbation selection). The secondary screen was performed in duplicate. Reproducibility in the EU phenotypes of the two replicate secondary screens was very high (Fig. 4i), with a Pearson’s correlation for mean residual mean EU of 0.98. When comparing mean residual mean EU between the secondary screen and the genome-wide screen (Fig. 4j), reproducibility was still very high (Pearson’s correlation 0.82), despite these screen being performed 9 months apart. As shown in Fig. 4k, correlations for other EU phenotypes measured in the two screens, including those quantifying nucleolar and non-nucleolar EU contributions to nascent RNA production were also high (0.78–0.82).

## Usage Notes

The first few plates show very high intensities for SE, which we hypothesize is due to the freshly dissolved succinimidyl ester (SE) at the start of the screen. However, this artefact is completely removed by normalizing the data to the scrambled siRNA control wells on each plate (Fig. 4c). Wells that may be subject to liquid-handling errors in dispensing transfection reagent were on plates 62, 65, 67, 68, 69, 70, 71, as discussed in “Uniformity of replicate controls in genome-wide screen”. The library file provided in the IDR data record contains this information as a quality control annotation^11^. We expect that these plates contain a low percentage of false negative wells because only a small subset of KIF11 controls on these plates were non-transfected (Fig. 4f) and the vast majority of SLC25A3 controls showed the expected RNA production rate increase seen across the screen (Fig. 4g).

## Data Availability

All image processing and extraction of quantitative measurements was done using the TissueMAPS framework, an open-source software project developed in our lab (https://github.com/pelkmanslab/TissueMAPS). In addition to the detailed description of the image processing workflow in Table 1, parameter settings files are provided in the data record (IDR, idr0093)^11^. Using these parameter settings combined with the open-source code of TissueMaps allows for reproduction of the exact image analysis pipeline. Additionally, the Ilastik project used for the nucleolar pixel classification is also provided in the data record (IDR, idr0093). Custom code used to computationally scale our analysis to the required data volume is specialised for our computing architecture and therefore not provided here.

## Online-only Table

**Online-only Table 1:** List of reagents and concentrations.

Type	Reagent	Solvent	Stock concentration	Final concentration	Source	Identifier
Seeding, Transfection, EU pulse and Fixation						
Cell culture plate	384-well Greiner uClear plates				Greiner Bio-One	781092
Media	DMEM (high glucose)				Thermo Fisher	41965062
Media supplement	GlutaMAX		100%	1%	ThermoFisher	35050038
Media supplement	Foetal Calf Serum		100%	10%	Sigma-Aldrich	F7524-500ML Lot FBS BCBQ7890V
Reduced Serum Media	OptiMEM				Thermo Fisher	11058021
Transfection reagent	RNAi-MAX	OptiMem	1	1:180	Thermo Fisher	13778075
Metabolic labelling of RNA	EU	Complete growth media	100 mM	1 mM	Baseclick	BCN-003-100
Fixation reagent	PFA	PBS	16%	4%	Electron Microscopy Sciences	15710
siRNA Library and Controls						
siRNA	siRNA Silencer Select Human Genome Wide Library	H_2_0	5 µM	5 nM	Silencer Select, ThermoFisher	4397926
siRNA	Negative control (Scrambled)	H_2_0	540 nM	5 nM	ThermoFisher	4390843
siRNA	KIF11	H_2_0	540 nM	5 nM	ThermoFisher	s7902
siRNA	PIM2	H_2_0	540 nM	5 nM (total)	ThermoFisher	s21749, s21750, s21751
siRNA	SLC25A3	H_2_0	540 nM	5 nM (total)	ThermoFisher	s10427, s10428, s10429
siRNA	LRRK2	H_2_0	540 nM	5 nM (total)	ThermoFisher	s42413, s42414, s42415
siRNA	NUDT4	H_2_0	540 nM	5 nM (total)	ThermoFisher	s22020, s22021, s22022
siRNA	SBF2	H_2_0	540 nM	5 nM (total)	ThermoFisher	s37818, s37819, s37820
siRNA	GRIP2	H_2_0	540 nM	5 nM (total)	ThermoFisher	s37445, s37446, s37447
Click reaction						
Buffer	Tris-buffered saline (TBS)			50 mM Tris pH 8.0, 150 mM NaCl		
Fluorophore	Azide Alexa Fluor 488	DMSO	10 mM	5uM	ThermoFisher	A10266
Reducing agent	Sodium Ascorbate	TBS	1 M	100 mM	Sigma-Aldrich	A7631
Catalyst	CuSO4	H_2_0	500 mM	2 mM	Sigma-Aldrich	7758-98-7
Immunofluorescence and other stainings						
Permeabilization reagent	Triton X-100	PBS	1	0.5%	Sigma-Aldrich	T8787
Blocking reagent	BSA	PBS		1%	Abcam	ab7475
Primary Antibody	Anti-PCNA monoclonal antibody (rabbit) (clone: D3H8P)	1% BSA	1	1:800	Cell Signaling Technology	13110
Secondary Antibody	Anti-rabbit IgG polyclonal antibody (goat) Alexa Fluor-568	1% BSA	1	1:500	ThermoFisher	A-11011
DNA staining	DAPI	H_2_0	1	1:270	ThermoFisher	D1306
Total protein staining	Alexa Fluor 647 NHS Ester	50 mM Carbonate Buffer pH 9.2	1	1:60000	ThermoFisher	A20006
Antibiotic	Penicillin-Streptomycin	PBS	1:50	1:100	ThermoFisher	15140-122
