# Author Correction: High content genome-wide siRNA screen to investigate the coordination of cell size and RNA production

**DOI:** 10.1038/s41597-021-01096-2

**Published:** 2021-12-02

**Authors:** Micha Müller, Merve Avar, Daniel Heinzer, Marc Emmenegger, Adriano Aguzzi, Lucas Pelkmans, Scott Berry

**Affiliations:** 1 Department of Molecular Life Sciences, University of Zurich, Zürich, Switzerland; 2 Institute of Neuropathology, University of Zurich, Zurich, Switzerland

**Keywords:** RNA metabolism, High-throughput screening

Correction to: *Scientific Data* 10.1038/s41597-021-00944-5, published online 28 June 2021

The original version of this Data Descriptor omitted the following authors from the Author List: Merve Avar, Daniel Heinzer, Marc Emmenegger and Adriano Aguzzi. The Acknowledgement and Author Contributions sections have been updated to reflect the changes in the author list. These errors have now been corrected in both the PDF and HTML versions of the Article.

